# Development of a High-Efficiency Z-Form Selector for Single Crystal Blades and Corresponding Grain Selection Mechanism

**DOI:** 10.3390/ma12050780

**Published:** 2019-03-07

**Authors:** Xintao Zhu, Fu Wang, Dexin Ma, Andreas Bührig-Polaczek

**Affiliations:** 1 Foundry Institute, RWTH Aachen University, Intzestrasse 5, 52072 Aachen, Germany; zhudb8@gmail.com (X.Z.); d.ma@gi.rwth-aachen.de (D.M.); sekretariat@gi.rwth-aachen.de (A.B.-P.); 2 State Key Laboratory for Manufacturing System Engineering, School of Mechanical Engineering, Xi’an Jiaotong University, Xi’an 710049, China

**Keywords:** two-dimensional selector, grain separation, directional freezing, single-crystal element, super alloy

## Abstract

Single crystal (SX) is widely used in modern turbine blades to improve the creep fracture, fatigue, oxidation, and coating properties of the turbine, so that the turbine engine has excellent performance and durability. In this paper, the single crystal super alloy MM247LC is used as the research material. The evolution of grain structure in a two-dimensional grain selector was studied by directional experiments, and the mechanism of grain selection in the two-dimensional channel during directional solidification was clarified. In order to optimize the production process of single crystal turbine blades, the effects of the geometrical structure of a Z-type separator (i.e., wire diameter and take-off angle) on the crystal orientation, microstructure, and grain efficiency of blades were discussed.

## 1. Introduction

Single crystal super alloys, especially nickel-based super alloys, are widely used in the manufacture of engine blades due to their excellent creep resistance and high temperature strength [1,2,3]. In general, the resulting single crystal structure and Bridgeman furnace using a directional solidification processes, which are the most commonly used industrial products and single crystal products studied. In this process, the single grain structure is grown from <001>, which is the preferred orientation. In addition, the grain boundary, which reduces creep property, can be eliminated [4,5].

To achieve this process, grain selectors are usually used. With these, there is only one grain to be chosen from. A granulator usually consists of three parts: the starter block, the connecting part, and the granulator part. Some studies [6,7,8,9] have shown that the geometric shape of the grain selector has an important influence on the selection efficiency of grains and the orientation of grains. Among them, a continuous helical structure of helical selectors is the most widely used because of its high efficiency [10]. However, due to the complex three-dimensional (3-D) shape of the helical part, some geometric errors may still occur due to sudden failure.

**Figure 1:**
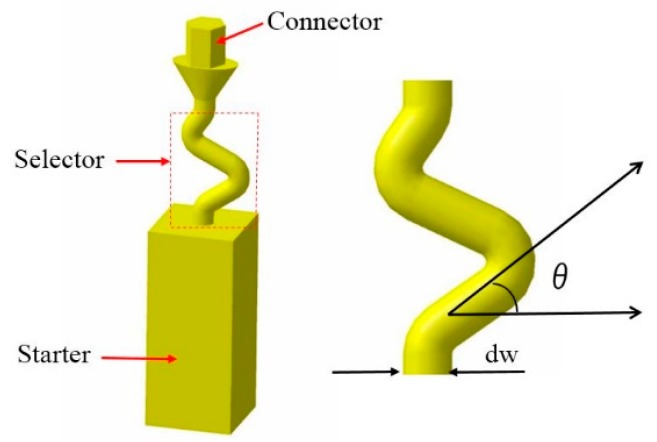
Schematic illustrations of the new 2-D grain selector and the corresponding grain selector portion.

Due to the difficulty in studying and analyzing 3-D helical pattern selectors and the possibility of using simplified 2-D projection of 3-D structures, we designed Z-form grain and various geometric parameter selectors (line diameter and take-off angle) to use 3-D printing technology to optimize the corresponding 2-D texture selectors of geometric pattern. A series of Bridgeman industrial experiments were carried out in the foundry institute of Aachen University, which demonstrated the physical process, as shown in Figure 1. The influence of selected geometry on grain selection was quantitatively analyzed by using scanning electron microscopy (SEM) equipped with electron backscattering diffraction (EBSD) and optical microscopy (OM). On this basis, the influence of the thickness and take-off angle of the selector on the experimental results is discussed, and the mechanism of the influence on the experimental results is proposed.

## 2. Experiments

### 2.1. Model Design of 2-D Grain Selector

Figure 1 shows a schematic of the new two-dimensional selector and the corresponding selector section. New options for three sections include the starter block, Z-form selector section, and connector section. A 10 × 10 × 30 mm^3^ (length × width × height) starter block and different angle of departure (theta equals: 15°–55°) and thickness (dw) = 0.18–0.54 cm characterize the selector section (Table 1 and Table 2).

### 2.2. Directional Solidification Experiments

The study uses the superalloy CM247LC, and the corresponding chemical composition is listed in Table 3.

The first step in the investment casting was to produce shell molds. In detail, the wax models of the 2-D selectors and ∅20 mm × 150 mm cylindrical bars were combined together with different parameters, these having been applied to investigate the effects of the geometry of the grain selector on grain selection.

**Figure 2:**
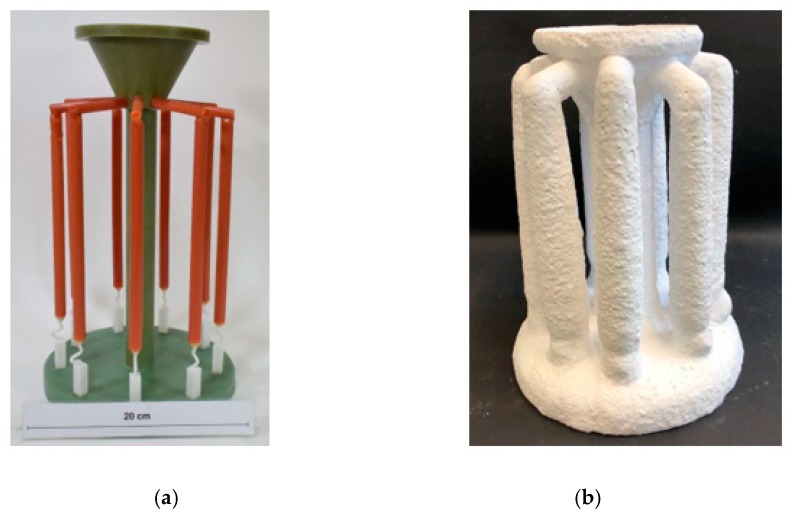
Images of the wax model (**a**) and the shell mold (**b**).

Figure 2a shows two parts of the model: the center bar and 10 elements. These units were assembled around the rods to form a cluster of wax molds. Then, the wax components were immersed in water-based ceramic slurries of different viscosities and gelatinized with alumina sand of different sizes. The integrated wax model was immersed in corundum paste (front coating and back coating) 12 times until the shell thickness reached 7 mm. After drying, the shell mold was dewaxed and further heated by steam. The remaining wax was then removed and the wax mold strengthened.

### 2.3. Microstructural Characterization

The sample was sandblasted to remove ceramic fragments still attached to its surface. After cleaning the sample, the selector was cut and polished, and the microstructure was analyzed. The 60 mL C_2_H_5_OH + 40 mL hydrochloric acid + 2 g (Cu_2_Cl·2H_2_O) etchant was used to reveal the microstructure and EBSD orientation.

## 3. Results and Discussion

### 3.1. Grain Structure Evolution in the Selector Part of the 2-D Selectors

**Figure 3:**
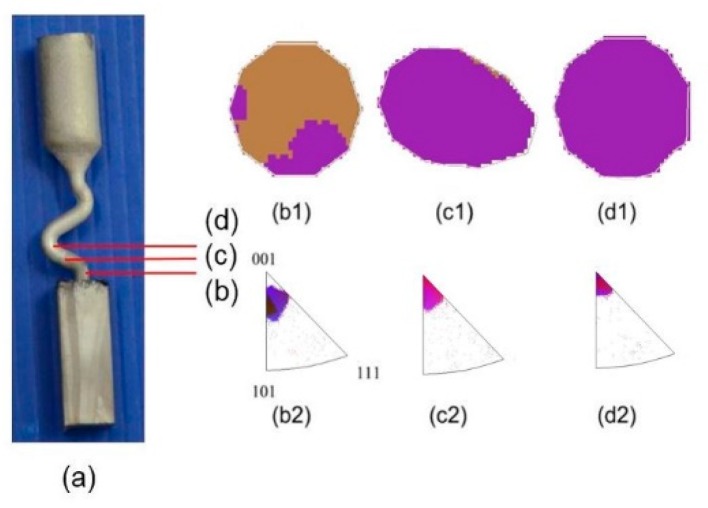
Experimental structure of a portion of the new selector with a thickness of 3 mm and a take-off angle of 40° (**a**); electron backscattering diffraction (EBSD) IPF-X (color code chosen along the growing direction) maps of the grain structure evolution (**b1**–**d1**) and the EBSD inverse pole figures in growth direction with various heights (**b2**–**d2**).

Figure 3 shows a typical example of the granular structure evolution of the 2-D selector with a thickness of 3 mm and a departure angle of 40°. Figure 3a illustrates the macro structure of the selector. Figure 3b1–d1 shows the characteristic microstructure at different heights transversally measured by EBSD along the selector (highlighted in Figure 3a). Figure 3b2–d2 shows the <001> reverse polarity diagrams (IPFs) corresponding to the Ni-based super alloy, indicating the grain orientation. It can be seen that the number of grains decreased significantly with the increase of height, but the grain size gradually increased (as shown in Figure 3b1–d1). At a height of about 36 mm, a single crystal was obtained. The large reduction in the number of grains in the part of the selector indicates that the two-dimensional selector has high efficiency in grain selection.

### 3.2. Effect of Selector Thickness on Grain Selection

**Figure 4:**
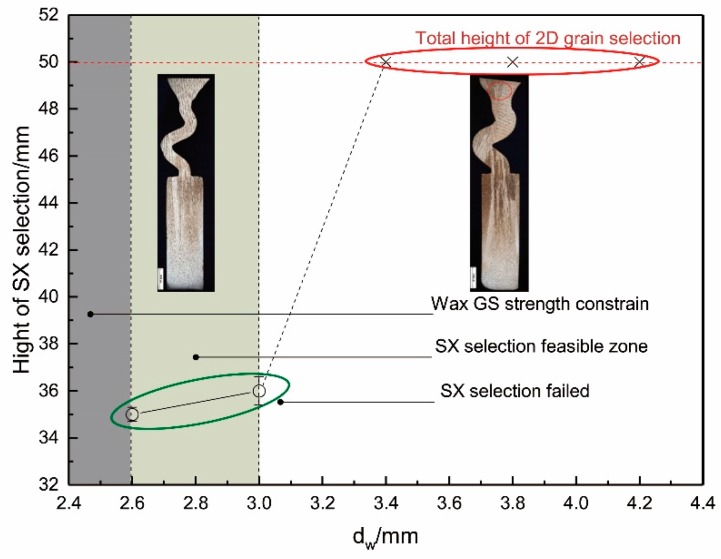
Experimentally observed heights of single crystal (SX) position versus selector thickness (d_w_).

The influence of particle selector thickness on selection is shown in Figure 4. Setting the take-off angle of 40° in the grain selection, obviously, when the selector is increased from 2.6 mm to 3 mm thick, one can select a single crystal, and the height of the SX structure decreases. However, as the selector thickness increases beyond 3 mm, the thicker selector cannot select SX. In addition, when the thickness of the selector decreases to less than 2.6 mm, the mold casting is prone to wax deformation due to the low strength of the selector, so this paper does not study selectors with thicknesses less than 2.6 mm. Therefore, a 3 mm selector is recommended to ensure the stability of the new selector.

**Figure 5:**
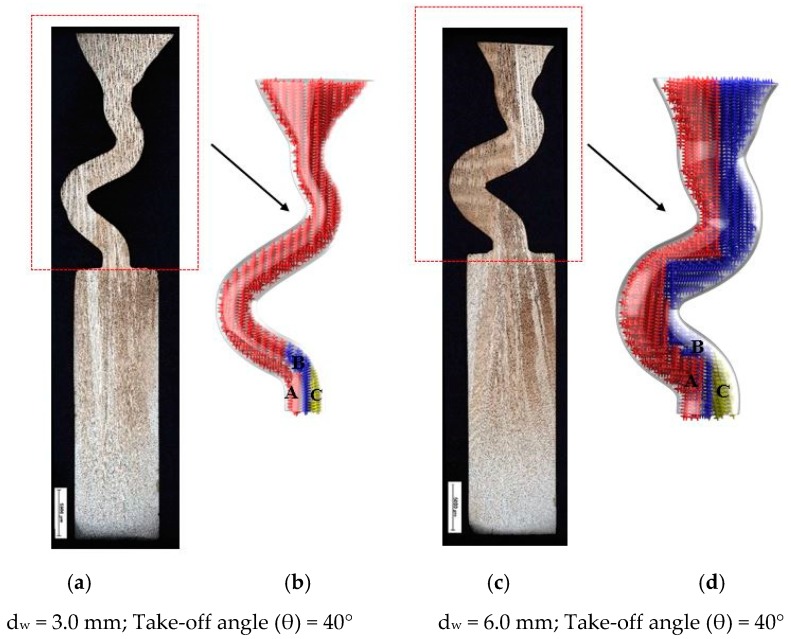
Illustration of the dendrite structure in the selector portion of the Z-form selectors with a diameter of 3 mm (**a**) and 6 mm (**c**) as well as their corresponding schematic diagrams (**b**) and (**d**).

As can be seen in Figure 5, there are three different kinds of grains growing in the selector part with diameters of 3 mm (b) and 6 mm (d). First, all three grains were planted at the same rate due to the withdrawal rate. Grain A has further room for growing in the horizontal direction, while the horizontal growth of grain B is blocked by grain A, so grain B must keep growing vertically until it exceeds the barrier built by grain A. Grain C is totally overgrown by grain A and grain B. With increasing selector thickness (as shown in Figure 5b), dendrite growth of grain B have enough space, overcomes the shortages of dendrite growth. Therefore, both grain B and grain A can grow in the casting.

**Figure 6:**
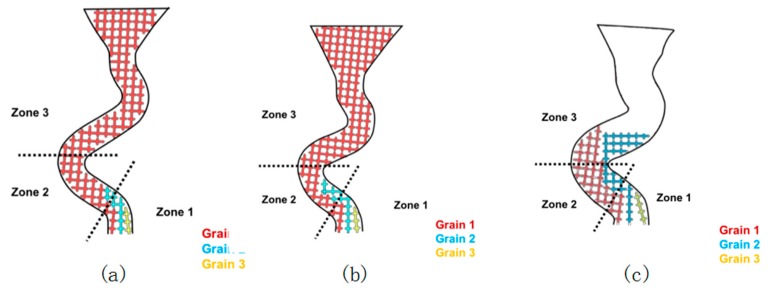
Schematic diagram of the grain selection process in Z-form grain selectors with different diameters: 2.6mm (**a**), 3.0mm (**b**), 6.0mm (**c**).

As shown in Figure 6, in zone 1, the vertical growth rate of the three particles was the same due to the same recovery rate. When the grain reaches the turning position in zone 1, there is still room for grain 1 to grow along the direction of the tunnel, while the growth of grain 2 is blocked by grain 1, so grain 2 must grow vertically until the blockage caused by grain 1 is overcome. Grain 3 is completely blocked by grain 1 and grain 2.

Figure 6a shows the schema of the grain selection process in a grain selector with a diameter less than 3 mm. It is clear that grain 1 grows horizontally with a higher velocity than grain 2. That means after grain 1 reaches the turning point of the selection tunnel, the growth front of grain 2 may still stay in the middle of zone 1, so there will be a great probability that the further growth of grain 2 is blocked by the tilt dendrite of grain 1. In that case, grain 2 stops growing before entering zone 2, and the increasing diameter (when it is under 3 mm) has little influence on the grain selection height. As a result, the grain selection height remains constant. 

Figure 6b shows the grain selection process of a grain selection machine with a diameter between 3 mm and 4.6 mm. In this case, grain 2 has a chance to reach zone 2, but due to the combined effect of the dendrite diameter of grain 1 and the rate difference between grain 1 and grain 2 (the dendrite diameter of grain 1 is large and cannot block the dendrite of grain 2), it is difficult for grain 2 to reach zone 3. The growth of grain 2 was blocked by the boundary of the selected channel, which could also explain the jump of the selected height of the grain when the diameter of the grain reached 3 mm. Then, with the increase of diameter (3–4.6 mm), the selected height of grains gradually increased, but did not jump to another height. This is because the growth of grain 2 was blocked by the boundary of the selected channel (the boundary between zones 2 and 3).

When the diameter of the selector is larger than 4.6 mm, as shown in Figure 6c, the boundary of the selected channel will no longer prevent the growth of the grain from zone 2 to zone 3. In other words, grain 1 and grain 2 both go into region 3. In this case, grain 2 grew vertically until the dendrite of grain 1 in zone 3 stopped growing. This means that when the diameter reaches 4.6 mm, the selected height of grain will jump again. Increasing the diameter has little influence on the selected height of grain, because grain 2 grows vertically in the tunnel in zone 3 and is eventually blocked by grain 1. However, when the diameter is large enough, grain 1 can no longer prevent grain 2 from growing into turbine blades. In this case, stray particles are formed.

To sum up, dendrites that deviate from the preferential dendrite can grow on the boundary of the selected part. The growth space of the spurious crystals is obviously larger than that of the smaller diameter selectors for the larger diameter selectors. Therefore, the spurious grains occupy the space of the preferential dendrites and grow together and stay in the bar, resulting in poor mechanical properties of the casting. For the selectors with smaller diameter, although the wall of the selectors provides a location for stray grains, there is no room for further growth of stray grains due to competition with priority dendrites that have not yet grown, which can prevent the defects of stray grains.

Although the results show that the effect of selecting the smaller diameter selector is better, in practical application, the weight of the bar will deform the selected part due to its weak stiffness. Considering the appearance of stray grains and the variation trend of deflection angle, the Z-type grain selector with a diameter of 3 mm was selected.

### 3.3. Effect of Take-Off Angle on Grain Selection

**Figure 7:**
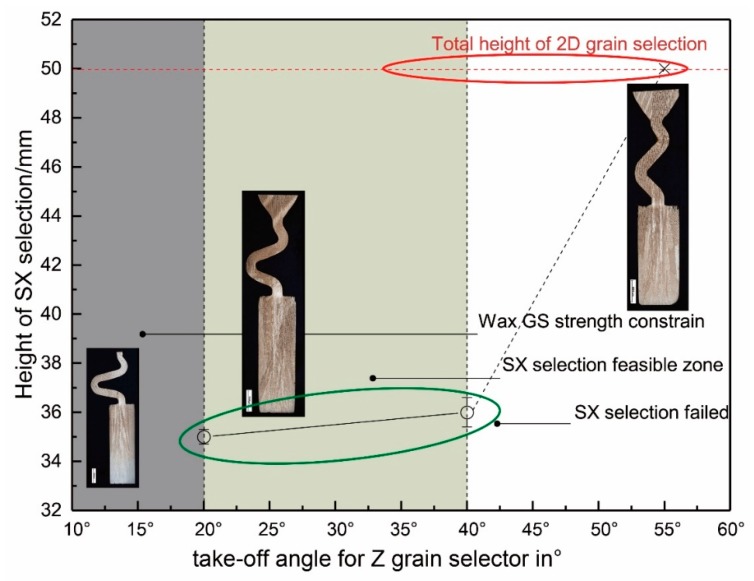
The relationship between heights of SX position and the take-off angle of the grain selector portion.

Figure 7 shows the influence of take-off angle on grain selection when the thickness of the selector is 3 mm. The SX can be chosen successfully with a take-off angle (theta) less than 40°. The selected height decreases with the decrease of take-off angle. The results show that with the decrease of the take-off angle, the selection efficiency of the two-dimensional selector is improved. However, when the angle of departure is less than 15°, the structure is unstable. The wax 2-D selector breaks easily when assembled with the casting into the die set. Considering the stability of the 2-D selector, it is recommended that the take-off angle be 40°.

**Figure 8:**
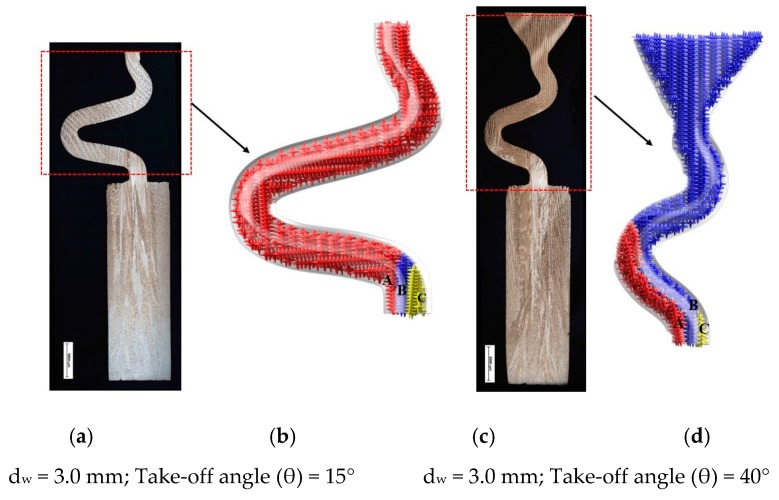
Optical images of the dendrite structure in the selector portion of the selectors with take-off angles of 15° (**a**,**b**) and 40° (**c**,**d**) as well as their corresponding schematic diagrams.

Figure 8 shows the dendrite structure part of the selector at take-off angles of 15° and 40° and the corresponding principle diagram. As can be seen, three grains grow from the starter block to the selector portion of the two-dimensional selector. Due to well consistent with the vertical temperature gradient and resulted lower undercooling, the dendrite tip of grain B grow faster than the dendrite tips of grains A and C. Similarly, the grain C is overgrown by grain B since its primary tips are impinged not only by the mold wall but also the dendrite trunks of grain B. When the selector’s take-off angle is big enough (Figure 8B), grains A and B can grow into the castings due to enough space or undercooling. However, when the take-off angle is reduced, the position of particle A receives better cooling conditions due to the suspended structure. The growth and branching rate of the dendrite arm of grain A is higher than that of grain B, which hinders the growth of grain B. Therefore, only grain A can survive and grow into casting, as shown in Figure 8A.

**Figure 9:**
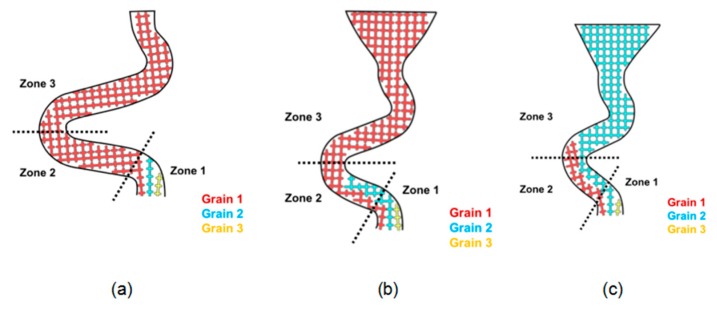
Schematic drawings of the grain growth process in Z-form grain selectors with various take-off angles: (**a**) 15°, (**b**) 30°, (**c**) 40°.

Figure 9a,b shows that, with increasing take-off angles, the heat flow becomes homogeneous resulting in a smaller difference in temperature between grain 1 and grain 2. In other words, with the increase of the take-off angle, grain 2 can reach a higher position in zone 2 leading to an increasing grain selection height under 40°.

When the angle of departure finally reaches 40°, as shown in Figure 9c, grain 2 could finally reach area of grain 1 through the selection of the grain growth in tunnel. In this case, the growth of grain 1 was blocked vertically by the boundary of the selected tunnel, while the growth of grain 2 was blocked horizontally. The position of the grain 1 block is defined as the grain option height when a Z-form selectors take-off angle is 40°. A take-off angle of more than 40° results in stray grains.

To sum up, dendrites that deviate from the preferential dendrite can grow on the boundary of the selected part. The larger the take-off angle of the selector, the growth space along the growth direction deviates from the crystal is much larger than the smaller the take-off angle of the selector; therefore, the stray grain occupies the space of preferential dendrites, so they grow together and stay in the rod, so the mechanical properties of the cast component will be greater than that of the single crystal component. The selectors with smaller take-off angles, although the selectors walls provide the location of stray grains, do not favor dendritic growth due to competition, and the further growth of stray grains was hindered, because there are no more spatial barriers to select parts, and stray grain defects are preventable.

The results prove that it is better to choose selector with a smaller take-off angle, but in the reality, the weight of the rod will deform the selection part due to its weak rigidness.

## 4. Conclusions

Using an optical microscope and EBSD technique, grain selection in a two-dimensional solidification process was studied systematically. The EBSD technique was used to study the grain selection under different geometric parameters. The purpose of the Z-type selector is only to select a single grain, and the efficiency of the selector largely depends on the thickness and the departure angle. The experimental results show that the whole grain selection efficiency is higher when (a) the take-off angle is smaller, and (b) the thickness is smaller. The stability of the 2-D texture selector in the casting, a thickness of 3 mm, and a Z-form departure angle of 40° form the optimal parameters of grain selectors. The selection of grains in the solidification process of a new type of two-dimensional selector is mainly affected by geometric blockage and local thermal conditions.

## Figures and Tables

**Table 1:** Variation in thickness of the grain selector portion.

Z-Form Grain Selector with Various Diameter	Single Crystal				Stray Grain			
	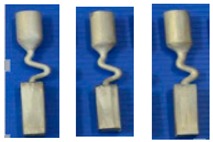				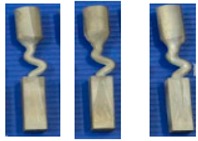			
Stray Grain	No				Yes			
Probe	Zd1	Zd2	Zd3	Zd4	Zd5	Zd6	Zd7	Zd8
Diameter (cm)	0.18	0.22	0.26	0.30	0.38	0.42	0.46	0.54

**Table 2:** Variation in take-off angle of the grain selector portion.

Z-Form Grain Selector with Various Take-Off Angle	Single Crystal						Stray Grain		
	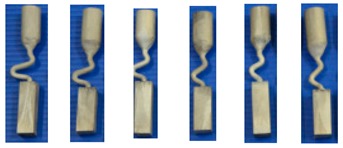						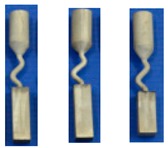		
Stray Grain	No						Yes		
Sample	Z1	Z2	Z3	Z4	Z5	Z6	Z7	Z8	Z9
Take-Off angle	15°	20°	25°	30°	35°	40°	45°	50°	55°

**Table 3:** The chemical composition of superalloy CM247LC (wt.%).

Elements	Al	Ti	Cr	Mo	Co	W	Ta	Hf	C	Ni
wt.%	5.49	0.74	8.03	0.5	9.41	9.87	2.9	1.36	0.094	Bal.
